# Age and sex, but not depression or anxiety, predict P3 amplitude during adolescence

**DOI:** 10.1016/j.dcn.2025.101640

**Published:** 2025-11-01

**Authors:** Marta Korom, Marco McSweeney, Selin Zeytinoglu, Lucrezia Liuzzi, Daniel S. Pine, Nathan A. Fox, Katharina Kircanski

**Affiliations:** a National Institute of Mental Health, Bethesda, MD, United States; b University of Maryland, College Park, MD, United States

**Keywords:** P3 amplitude, Depression, Anxiety, Age, Sex

## Abstract

Reduced P3 amplitude during selective attention has been linked to depression in cross-sectional studies primarily with adults. Neurodevelopmental research has yet to examine relations between age-related changes in P3 amplitude, assessed across multiple time points, and the emergence of depressive and anxiety symptoms during adolescence, which may vary by sex. The present study addresses this gap by testing the effects of between- and within-person depressive symptoms, age, and sex on P3 amplitude during the Flanker task, across up to three age time points in a sample of adolescents (N = 190, ages ∼12, 15 and 18) at risk for developing internalizing symptoms. When depression was measured continuously without adjusting for age and sex, higher within-person depressive symptoms emerged as a significant predictor of reduced P3 amplitude. However, when age, sex, and depression (continuous or binary diagnostic status) were modeled together, only age and sex, but not depression, remained significant predictors of P3 amplitude. Specifically, P3 amplitude decreased with age, and males consistently exhibited higher P3 amplitudes than females, with stable age-related decrease across sexes. For anxiety, neither between- nor within-person symptoms were significantly associated with P3 amplitude, with or without age and sex included in the model. Similar to the findings for depression, however, age and sex were significant predictors of P3 amplitude. Thus, previous studies involving a single assessment of P3 amplitude and depression symptoms may be influenced by developmental factors.

## 1 Introduction

The amplitude of the P3 event-related potential (ERP) provides a direct measure of attention-driven updating of existing schema related to new information (Donchin, 1981, Polich, 2007, Riggins and Scott, 2019). P3 amplitude is commonly measured using selective attention tasks, like oddball paradigms (Polich, 2007) and the Flanker task (Eriksen and Eriksen, 1974, Eriksen, 1995), which require the allocation of attentional resources to infrequent or target stimuli among distractors (Riggins and Scott, 2019). Between-subject variability in P3 amplitude has been linked to various factors. For example, across a variety of task paradigms, relatively high P3 amplitude relates to enhanced information processing abilities, whereas reduced P3 amplitude relates to poor cognitive performance. For instance, reduced P3 amplitude has been associated with worse inhibitory control on the Stop Signal Reaction Time Paradigm (Brennan and Baskin-Sommers, 2018), attentional processes on auditory oddball paradigms (Szuromi et al., 2010, Tsai et al., 2012), and working memory on the n-back test (Saliasi et al., 2013). Additionally, lower P3 amplitude correlates with mood disorders in adults on oddball paradigms (Bruder et al., 2009, Röschke and Wagner, 2003), monetary reward tasks (Klawohn et al., 2022), and the Flanker task (Santopetro et al., 2021), taken to support the hypothesis that disruptions in cognitive processes shape the pathogenesis of depression.

While the adult literature linking reduced P3 amplitude with depressive symptoms is rich and relatively consistent (Kangas et al., 2022), less is known about pediatric populations. To date, eight studies have examined the correlation between P3 amplitude on selective attention tasks and depressive symptoms in youth, yielding mixed results. Specifically, three studies using the Flanker Task (Santopetro et al., 2020, Santopetro et al., 2021, Santopetro et al., 2022) and another using a visual oddball paradigm (Houston et al., 2003) found reduced stimulus-locked P3 amplitude in youth meeting diagnostic criteria for depression or with higher depressive symptoms as compared to youth with no or low depressive symptoms. Furthermore, one study, utilizing an auditory oddball paradigm, found the opposite effect (Lepistö et al., 2004), and three studies using similar auditory oddball paradigms found no significant associations with depressive symptoms or diagnoses (Feldmann et al., 2018, Greimel et al., 2015; Houston et al., 2004). Taken together, results from these eight studies suggest that the stimulus-locked P3 amplitude on the Flanker Task may be uniquely sensitive to neurophysiological correlates of depression. Importantly, no studies to date have examined how changes in both stimulus-locked P3 amplitude across repeated assessments and depressive symptoms unfold together across adolescence. While some research has assessed the prospective, longitudinal association between a single baseline measure of P3 amplitude and later depressive symptoms (Santopetro et al., 2020), to the best of our knowledge, there have been no published studies with repeated assessments of both P3 amplitude and depressive symptoms over time. This limits our understanding of how neurodevelopmental changes in P3 amplitude relate to the emergence and progression of depressive symptoms during adolescence – a key period for the emergence of depression particularly in girls.

Age and sex are two important factors influencing both P3 amplitude and depressive outcomes (Dinteren et al., 2014, Melynyte et al., 2018, Riggins and Scott, 2019). Although longitudinal research is limited, cross-sectional studies have provided some insight into the developmental changes in P3 amplitude (Ehlers et al., 2001, Polich et al., 1990). Several reports suggest an increase in P3 amplitude across childhood and adolescence (e.g., Polich et al., 1990; Tsai et al., 2012), whereas other studies report no age effects (e.g., Ehlers et al., 2001) or age-related reduction in P3 amplitude during this period (Pfueller et al., 2011). A non-linear, convex relationship between age and P3 amplitude has also been observed in large cross-sectional studies, where the P3 amplitude increases during childhood, plateaus in adolescence and young adulthood, and then decreases in later years (Dinteren et al., 2014). Such a pattern may reflect age-related cognitive decline during adulthood (Ashford et al., 2011, Rossini et al., 2007, Walhovd et al., 2008). As adolescence is a sensitive period for both brain development (Fuhrmann et al., 2015) and the onset of depression (Andersen and Teicher, 2008, Thapar et al., 2012), which may be linked to a decrease in P3 amplitude, it is critical to account for normative age-related changes in P3 amplitude when examining longitudinal associations between depressive symptoms and P3 amplitude. Previous research has generally examined the effects of age and depression on P3 amplitude independently, underscoring the need for more integrative studies.

### 1.1 Study goals and hypotheses

The primary goal of the present study was to address this gap in the literature by examining how depressive symptoms, age, and sex predict stimulus-locked P3 amplitude in a sample of adolescents at risk for developing emotional disorders, assessed at years 12, 15, and 18. Based on prior empirical work (Houston et al., 2003, Santopetro et al., 2020, Santopetro et al., 2021, 2022; Thompson et al., 2025), we hypothesized that age would show a positive association with P3 amplitude, whereas depressive symptoms would have a negative relationship with P3 amplitude over time, suggesting an additive relation between age and depression in shaping P3 amplitude. A secondary goal was to conduct parallel analyses with anxiety instead of depression. Considering the high comorbidity between depression and anxiety (Kalin, 2020), we aimed to test whether heightened depressive symptoms are uniquely associated with P3 amplitude, or if both anxiety and depression are predictive of P3 amplitude. Based on Santopetro and colleagues’ (2021) work, we hypothesized that higher depressive, but not anxiety symptoms would be associated with blunted P3 amplitude over time. Furthermore, given the significant sex differences in depression prevalence, with females at higher risk than males (Salk, Hyde, and Abramson, 2017), an exploratory aim was to test whether sex moderates the posited relationship between P3 amplitude and depressive symptoms. Specifically, we expected the negative association between depression and P3 amplitude to be stronger in females compared to males, while controlling for normative age-related changes in P3 amplitude (Santopetro, Kallen, et al., 2021).

Supplementary analyses were completed to explore bidirectional associations between P3 amplitude, depression, and anxiety using random-intercept cross-lag modeling (RI-CLPM; see the results in *Section 1* of the Supplementary materials). Finally, we repeated all of the primary analyses with P3 amplitude as the predictor and depression and anxiety as outcome variables. The results of these supplementary analyses are available in *Section 2* of the Supplementary materials.

## 2 Methods

### 2.1 Participants

Participants were adolescents enrolled in a longitudinal study examining the impact of infant temperamental risk on the development of anxiety, with usable EEG data collected during the adolescence assessments. Participants were originally selected based upon their patterns of reactivity to novel auditory and visual stimuli at 4 months of age and were followed throughout young adulthood. Seven hundred and seventy-nine infants were recruited from the Washington, DC metro area for a study of early temperament, with the aim of identifying and recruiting infants showing distinct temperamental reactivity patterns to novelty. Infants were excluded if they were born premature, had low birth weight, a known developmental disorder, and/or experienced birth complications. Parental consent was obtained prior to all visits and child assent was obtained prior to the 10‑ and 12‑year visits. All 779 infants recruited were screened at four months using an assessment of reactivity to novel visual and auditory stimuli according to the procedure described in Calkins et al. (1996). Infants’ behaviors were coded for the frequency of positive affect, negative affect, and motor reactivity. The scores of the first 96 infants enrolled were used to generate cut‑off criteria: infants above the median for both positive affect and motor reactivity were classified as “positive reactive,” whereas infants above the median for both negative affect and motor reactivity were classified as “negative reactive.” When infants met criteria for both groups, they were sorted into “high positive reactive” or “high negative reactive” according to their affective bias, i.e., the difference between their standardized positive and negative affect scores; infants whose affect was more positive than negative were classified as high positive reactive, and vice versa. Those not meeting criteria for either group were classified as “non‑reactive.” This screening procedure identified 291 infants—mean age 4 months and 2 days—who were invited to participate in subsequent longitudinal assessments. These 291 infants were oversampled for temperamental reactivity (106 positive reactive, 116 negative reactive, and 69 non‑reactive). Parents and adult participants signed an informed consent and adolescents signed an assent form at each wave, and all procedures were approved by the University of Maryland’s Institutional Review Board.

During the adolescent follow-up, 212 participants took part in the EEG assessment. Twelve were excluded due to significant noise in the data, six due to ocular artifacts, two because of poor brain response at the Pz electrode, and two due to having fewer than 30 correct trials and an overall task accuracy 3 SD below the group average on the Flanker task (Barker et al., 2021), yielding a final sample of 190 participants with at least one usable EEG data point. The attrition rate from infancy (291 participants) to the adolescent assessments (190 participants) was 34.7 %, which is comparable to (Launes et al., 2014, Teixeira et al., 2021) or lower (Gustavson et al., 2012) than attrition rates reported in other long-term birth cohort studies. To examine the possibility of selective attrition, we compared demographic variables at the time of enrollment in infancy, including sex, parental ethnicity, parental education at the time of enrollment) and early temperament between those who contributed any adolescent EEG data and those who did not. These analyses revealed no significant differences (sex: χ²(1, N = 291) = 1.32, p = .25; maternal ethnicity: χ²(4, N = 290) = 3.66, p = .45; missing N = 1; paternal ethnicity: χ²(4, N = 287) = 1.56, p = .82, missing N = 4; maternal education: χ²(3, N = 289) = 2.82, p = .42, missing N = 2; paternal education: χ²(3, N = 286) = 0.07, p = .99, missing = 5; temperament: χ²(2, N = 291) = 2.74, p = .254), suggesting that participation into adolescence was not systematically biased based on these key baseline variables. Participants who met inclusion criteria included 133 adolescents at the 12-year assessment (age range: 12.08–15.05 years), 137 at the 15-year assessment (age range: 15.02–17.85 years), and 108 participants at the 18-year assessment (age range: 17.65–19.87 years). Of these, 63 participants had one usable data point, 66 had two, and 61 had three usable data points. The sample was racially diverse and sex-balanced. Detailed demographic information, along with Chi-square tests and *t*-tests comparing the distribution of the demographic variables at the 12, 15, and 18-years visit, are provided in Table 1. These analyses yielded non-significant results (all p > .05).

**Table 1:** Descriptive statistics and average depression and anxiety scores stratified by age of data collection.

**Variable, N (%) or M (SD)**	**Age 12**	**Age 15**	**Age 18**	**Test Statistic**
Sex	Females, N = 68 (51.13 %) Males, N = 65 (48.87 %)	Females, N = 75 (54.74 %) Males, N = 62 (45.26 %)	Females, N = 64 (59.26 %) Males, N = 44 (40.74 %)	χ² = 1.59, p = .451
Minority status	30 (22.56 %)	33 (24.09 %)	28 (25.93 %)	χ² = .37, p = .83
Parental education	No high-school= 21 (15.79 %) High school = 56 (42.11 %) Univ. degree = 50 (37.59 %) Did not answer = 6 (4.51 %)	No high-school = 23 (16.79 %) High school = 58 (42.34 %) Univ. degree = 50 (36.5 %) Did not answer = 6 (4.38 %)	No high-school = 20 (18.52 %) High school = 40 (37.04 %) Univ. degree = 44 (40.74 %) Did not answer = 4 (3.7 %)	χ² = 1.18, p = .98
Age at EEG data collection	13.06 (0.53) years	16.01 (0.52) years	18.43 (0.55) years	
Age at CBCL^1^/ASR^2^ completion	13.05 (0.55) years	16.01 (0.53) years	19.26 (0.72) years	
Age at SCA(A)RED^3/4^ completion	13.10 (0.58) years	15.94 (0.52) years	19.26 (0.72) years	
Age at KSADS^5^/SCID^6^ completion	13.00 (0.65) years	16.27 (0.54) years	18.6 (0.32) years	
CBCL^1^/ASR^2^ score	1.34 (1.68)	2.43 (2.96)	6.32 (5.27)	
Anxiety score	16.92 (10.77)	20.35 (12.19)	22.82 (16.66)	
Depression diagnosis	1 (0.75 %)	17 (12.41 %)	31 (28.7 %)	

*Note.* Minority status - binary variable, parent report: 0 = belonging to the ethnic majority group and 1 = belonging to an ethnic minority group; ^1^CBCL - Child Behavior Checklist; ^2^ASR - Adult Self Report; ^3^SCARED - Screen for Child Anxiety Related Emotional Disorders; ^4^SCAARED - Screen for Adult Anxiety Related Emotional Disorders; ^5^KSADS - Kiddie Schedule for Affective Disorders and Schizophrenia; ^6^SCID - Structured Clinical Interview for DSM Disorders.

### 2.2 Procedures

Parents completed the consent forms prior to data collection at years 12 and 15, and participants consented to participation at age 18. We collected EEG data using the Flanker task at years 12-, 15-, and 18 in the laboratory. Questionnaires were completed using REDCap either during one of the visits or remotely. Semi-structured clinical interviews were conducted at all three time points either in-person or via Zoom. Participants received financial compensation for their involvement in the study.

### 2.3 Measures

#### 2.3.1 Continuous depression symptom scores

*Child Behavior Checklist (CBCL):* Depressive symptoms were assessed via parent report on the CBCL Affective Problems subscale at years 12 and 15 (Achenbach, 2000, Achenbach and Rescorla, 2014). The CBCL is a widely used instrument designed to assess a child’s behavioral and emotional functioning. It includes 113 items that measure internalizing and externalizing behaviors, such as depression, anxiety, aggression, and social problems. Parents rated how true each statement was on a 3-point scale (0 = not true, 1 = somewhat or sometimes true, 2 = very/often true), with higher scores indicating greater concerns. The internal consistency of the Affective Problems subscale was acceptable in the sample (year 12: α =.601; year 15: α =.772). Items were summed such that higher values indicate more depressive symptoms.

*Adult Self-Report (ASR):* Depressive symptoms were assessed via self report on the DSM-oriented Depressive Problems scale of the ASR at age 18 (Achenbach, 2011, Achenbach and Rescorla, 2014). The ASR is designed to assess emotional and behavioral functioning in adults 18 and older. It is the adult self-report version of the CBCL, with both tools assessing similar domains of functioning using the same 3-point scales. The ASR consists of 126 items that evaluate mental health and behavioral problems, including depressive symptoms. Four items assessing suicidal intent, and physical harm to oneself or others were removed from the questionnaire (items 18, 57, 91, 97), two of which were part of the Depressive Problems scale (items 18 and 91). Higher values indicated more depressive symptoms. The internal consistency of the Depressive Problems scale was good in the sample (α =.879).

#### 2.3.2 Diagnosis-based depression variables

*Kiddie Schedule for Affective Disorders and Schizophrenia (KSADS):* At years 12 and 15, trained clinicians completed the KSADS (Kaufman et al., 1997), a semi-structured diagnostic interview, with both adolescents and parents. For the purposes of the current study, we examined clinical diagnoses of unipolar depressive disorders. Specifically, participants were coded as meeting criteria for a depressive disorder if either the parent or the participant reported a history or current diagnosis of major depressive disorder (MDD) and/or dysthymia at the time of the assessment, following DSM‑5 criteria. Once a diagnosis was made, it was carried forward to future assessments.

*Structured Clinical Interview for DSM Disorders (SCID):* At age 18, trained clinicians completed the SCID-5 (First, 2015) with the study participants. The SCID is a widely used and validated semi-structured diagnostic interview designed to evaluate adult mental health disorders across a broad range, following DSM‑5 criteria. For this study, the same modules assessed in the KSADS (past or current MDD or dysthymia) were included.

#### 2.3.3 Continuous anxiety symptom scores

*Screen for Child Anxiety Related Emotional Disorders (SCARED):* The SCARED is a self-report questionnaire designed to assess anxiety symptoms in youth between 8 and 18 years of age (Birmaher et al., 1997). It consists of 41 items that assess the frequency of a range of anxiety disorders, including generalized anxiety disorder, separation anxiety disorder, social phobia, and specific phobias across different contexts. Youth are asked to decide how frequently they feel as described in the item on a 3-point scale (0 = Not True or Hardly Ever True, 1 = Somewhat True or Sometimes True, 2 = Very True or Often True). Items were summed and higher scores indicate more anxious symptoms. The internal consistency of the SCARED was good in the sample (year 12: α =.91; year 15: α =.90).

*Screen for Adult Anxiety Related Emotional Disorders (SCAARED):* The SCAARED is a self-report questionnaire designed to assess the frequency of anxious symptoms in adults (Angulo et al., 2017). It is the adult version of SCARED (Birmaher et al., 1997) consisting of 44 items, with a rating scale structured the same way as the SCARED. Items were summed and higher scores indicate more anxious symptoms. The internal consistency of the SCAARED was good in the sample (α =.96).

### 2.4 EEG data collection and analytical approach

#### 2.4.1 EEG procedures

The Flanker Task (Eriksen and Eriksen, 1974, Eriksen, 1995) assesses selective attention and response inhibition. Previous studies have used event-related potentials (ERPs) from this same Flanker dataset to examine various psychophysiologic indices, including error-related negativity and other ERPs (Buzzell et al., 2017, Buzzell et al., 2019, Conte et al., 2023, Liuzzi et al., 2023, McSweeney et al., 2021, Morales et al., 2022, Shner-Livne et al., 2024). However, none has examined the P3 amplitude component elicited during the Flanker task in longitudinal analyses or its association with mental health outcomes. All EEG recordings were conducted in a dimly lit and sound-attenuated room and all participants were alone in the room. The Flanker task included 12 blocks each consisting of 32 trials. Each trial began with the presentation of a fixation cross lasting 300–600 ms, followed by the presentation of a central arrowhead flanked on each side by two additional arrowheads facing either in the same direction (congruent, < < < < <, > > > >) or in the opposite direction (incongruent, < < > < <, > > < > >) for 200 ms, ending with a 1900 ms intertrial interval when participants viewed a blank screen. The proportion of congruent to incongruent trials was 1:1 (i.e., 50 % congruent, 50 % incongruent). Participants were instructed to ignore the flanking arrowheads and to indicate the direction of the central arrowhead by pressing a button on an EGI response pad button box (model: 4608150–50). At the end of each block, participants received feedback regarding their performance. If they performed at or below 75 %, text was presented on the screen indicating that they needed to be more accurate. If they performed at 90 % or higher, text was presented on the screen indicating that they needed to respond faster. If performance was between 75 % and 90 %, text was presented on the screen indicating that they were doing a good job. At all times participants were seated approximately 1 m from a 17” LCD monitor. The stimuli were presented using E-Prime 2.0.874 (Psychology Software Tools, Pittsburg, PA).

#### 2.4.2 EEG acquisition and preprocessing

EEG data were acquired using a 128-channel HydroCel Geodesic Sensor Net (Electrical Geodesic, Inc., Eugene, OR) connected to a Net Station Amps 300, sampled at 250 Hz and referenced online to the vertex electrode. Impedances were kept below 50 kΩ. The data were preprocessed following the procedures described in the Maryland Analysis of Developmental EEG (MADE) pipeline (Debnath et al., 2020) which utilizes the EEGLAB toolbox (Delorme and Makeig, 2004) and custom MATLAB scripts (The MathWorks, Natick, MA). In short, marker offsets were measured and corrected for the EGI system (constant 36 ms offset) and the E-Prime computer (15 ms stimulus related time offset). Continuous EEG data were then high-pass filtered offline at 0.3 Hz and low-pass filtered at 50 Hz. Bad channels were identified and removed using the EEGLAB plug-in FASTER (Nolan et al., 2010). To facilitate the removal of EEG artifacts, independent component analysis (ICA) was performed on copied individual EEG data sets with a 1 Hz high-pass filter. The finalized copied dataset was then segmented into 1 s epochs and noisy epochs were identified and removed if amplitudes exceeded ± 1000 µV or if power within the 20–40 Hz band was greater than 30 dB (after Fourier transform). If a channel contained artifacts in more than 20 % of the epochs, that channel was removed from both the copied dataset and the original dataset. ICA was then run on the copied dataset. ICA weights were subsequently applied back to the original dataset (Debener et al., 2010) and artifactual independent components were identified and removed using the Adjusted-ADJUST algorithm (Leach et al., 2020, Mognon et al., 2011).

Following the removal of artifactual activity, for ERP analyses, the continuous EEG data were segmented to −200 through 800 ms relative to Flanker stimulus onset and baseline corrected using the 200 ms period preceding stimulus onset. The data were then subjected to a final rejection procedure. To identify any remaining bad segments a voltage threshold of ± 125 µV was applied to the data. If more than 20 % of the remaining data was rejected, the offending bad channels were removed instead. Missing channels were then interpolated using the spherical spline method (Perrin et al., 1989) and the data were referenced to the average reference. Visual quality assessment of ocular artifacts was completed by a trained scientist and participants with ocular artifacts were excluded from the analytical sample. Information on the average number of interpolated channels during data preprocessing, omitted and anticipatory responses (responded in less than 150 ms), and artifact free congruent and incongruent correct trials per age group are available in Table 2.

**Table 2:** Flanker Task performance indices, stratified by data collection year.

**Age**	**Indices**	**All trials, M (SD)**	**Congruent Trials, M (SD)**	**Incongruent Trials, M (SD)**
**12 year**	Accuracy rate	84.06 (7.07)		
	Number of dropped trials (omitted or responses < 150 ms)	5.82 (12.16)		
	Number of interpolated channels	3.94 (1.89)		
	Number of correct trials		156.58 (29.91)	120.6 (28.66)
	Number of error trials		9.13 (8.07)	43.14 (20.48)
	Reaction time in ms		376.97 (48.63)	421.8 (62.65)
**15 year**	Accuracy rate	88.18 (4.73)		
	Number of dropped trials (omitted or responses < 150 ms)	1.43 (3.25)		
	Number of interpolated channels	3.78 (1.71)		
	Number of correct trials		180.65 (14.17)	141.71 (19.42)
	Number of error trials		3.09 (3.78)	40.25 (17.38)
	Reaction time in ms		353.23 (42.73)	406.7 (54.16)
**18 year**	Accuracy rate	89.38 (4.55)		
	Number of dropped trials (omitted or responses < 150 ms)	1.52 (5.87)		
	Number of interpolated channels	4.04 (1.69)		
	Number of correct trials		179.19 (13.62)	146.12 (16.48)
	Number of error trials		3.19 (4.16)	35.85 (16.18)
	Reaction time in ms		342.2 (30.9)	391.4 (39.38)

#### 2.4.3 P3 amplitude calculation

We calculated P3 amplitude separately for congruent and incongruent trials in which participants correctly responded to the stimulus, based on prior research indicating smaller P3 amplitudes in congruent trials compared to incongruent trials in pediatric populations (Faja et al., 2016, Johnstone and Galletta, 2013). Trial averaged ERPs for the congruent and incongruent conditions were first averaged over an 8 sensor cluster covering from Cz to Pz (electrodes 31, 54, 55, 61, 62, 78, 79 and 80 in the EGI 128-channel HydroCel Geodesic Sensor Net). The maximum positive peak in the 300–700ms post-stimulus time window was then determined with MATLAB’s *findpeaks* function, and P3 amplitude was estimated as the average voltage in the 100 ms window around that peak. This adaptive mean scoring approach was chosen to improve reliability by accounting for between-person variability in peak latency, particularly pronounced in developmental data (Clayson, Baldwin, and Larson, 2012). If multiple peaks were detected for a given participant and condition, the algorithm selected the most prominent peak within the 300–700 ms window of interest for the congruent and incongruent trials separately at any of the 8 electrodes noted above, covering the area from Cz to Pz.

### 2.5 Data modeling approach

We used R (Version 4.3.1; R Core Team, 2023) for data analysis and visualization. Prior to data modeling, age was grand mean-centered to improve interpretability of the findings, and the between- and within-person effects of depression were separated using the bmlm package in R (Vuorre and Bolger, 2018). The separation reflects conceptually distinct levels of variation in the continuous depressive symptoms: the between-person effect, representing each individual’s overall, trait-like mean level of depression across the three assessment waves, and the within-person effect, reflecting time‑specific deviations from that personal average during adolescence. By including both components in our mixed‑effects model, we capture stable inter-individual differences as well as dynamic intra-individual fluctuations—ensuring that associations with P3 amplitude are not misinterpreted through conflated time‑invariant and time‑variant influences (Curran and Bauer, 2011, Hoffman and Stawski, 2009). Next, we conducted preliminary analyses, including missingness analysis using Little’s Missing Completely at Random (MCAR) test, reliability testing of P3 amplitude across the three time points using zero-order correlations, and intraclass correlation (ICC) calculation for the P3 amplitude. In all analyses, P3 amplitude was modeled separately for congruent and incongruent correct trials as the outcome variable. This analytical decision was based on prior research reporting larger P3 amplitude in incongruent trials compared to congruent trials in pediatric samples, which we also replicated in the present study (incongruent > congruent: *β* = 0.88, *t*(560.54) = 5.82, *p* < .001, *CI* 95 %[0.58, 1.18]). Age, sex, and measures of depressive and anxious symptoms were the predictors of interest.

Mixed-effects modeling was used in R (using the *lmerTest* package; Kuznetsova et al., 2017) for hypothesis testing, which allows to account for repeated assessments nested within individuals. A random intercept was included in all models to allow the intercept to vary between participants. To determine if random slopes should be included for the time-varying predictors, we empirically compared the models with and without random slopes and used AIC, BIC, Likelihood Ratio Tests, and the Chi-squared test of model fit and statistical significance to assess the best-fitting model. Model comparisons suggested that the random slopes did not improve model fit in all but one model. Given that including the random slope in Model 1 did not change the model results compared to the random intercept only model, all time-varying predictors were modeled as fixed effects across all models (see detailed information in Section 3 of the Supplementary materials). To estimate degrees of freedom and calculate two-tailed *p*-values, Satterthwaite approximations were used in all models. All analyses were considered statistically significant if *p* < .05. Missing data were handled using Full Information Maximum Likelihood (FIML), which allows for the inclusion of all available data without excluding cases with missing values on any variables (Enders, 2022).

In our initial model examining developmental processes, we modeled sex and age as main effects, along with their interaction terms, to predict P3 amplitude. The between-person effect of sex-related changes in P3 amplitude refers to the differences in P3 ERP responses between males and females over adolescence. Specifically, this effect investigates whether the pattern of change across age differs by sex. Next, we modeled the between- and within-person effects of the continuous depression symptom score (CBCL at ages 12 and 15, and ASR at age 18) and depression diagnosis (KSADS at ages 12 and 15 and SCID at age 18) as the sole predictors of P3 amplitude. Then we combined the developmental and depression models to examine the additive effects of age, sex, between- and within-person depression score effects on P3 amplitude. We then repeated this model using the binary depression diagnosis as a predictor. Last, we modeled the between- and within-person effects of anxiety as the sole predictor of P3 amplitude, followed by a combined developmental model that modeled age and sex together with the between- and within-person anxiety effects on P3 amplitude.

We selected P3 amplitude as our outcome of interest because it has been conceptualized as a potential biomarker of depression risk in prior cross-sectional or limited prospective designs, despite the limitation that directionality cannot be established in such designs. In addition, the developmental trajectory of P3 amplitude is of intrinsic interest, as it reflects underlying neurodevelopmental processes; to our knowledge, this is the first study to examine such trajectories in a longitudinal framework. Nevertheless, in light of prior research, it also is important to examine directional effects for P3 amplitude. Accordingly, we also tested the reverse model with P3 amplitude as the predictor of depressive symptoms and examined bidirectional associations using random-intercept cross-lagged panel models (RI-CLPM). Since the main conclusion from these two additional analyses are similar to the conclusion from the analysis with depressive symptoms as a predictor, we only mention these results briefly. However, full details of these supplementary analyses appear in Section 1 and 2 of the Supplementary Materials. Finally, we conducted secondary analyses using P3 peak amplitude at the electrode site where the P3 amplitude component was maximal, rather than averaging across sites. As in the primary analyses, we modeled both between- and within-person effects of continuous depression (CBCL/ASR) and anxiety symptoms (SCA(A)RED), as well as binary depression diagnoses (KSADS/SCID).

## 3 Results

### 3.1 Missingness analyses

The *LittleMCAR* function from the BaylorEdPsych (Beaujean, 2012) package was used for missingness analyses (Little, 1988). We included medication use (missing: 12-year: N = 6, 15-year: N = 6, 18-year: N = 4), CBCL/ASR (missing: 12-year: N = 4, 15-year: N = 1, 18-year: N = 11), KSADS/SCID (missing: 12-year: N = 30, 15-year: N = 29, 18-year: N = 14), and SCARED (missing: 12-year: N = 2, 15-year: N = 2) variables from all data collection ages in the missingness analyses. Age, P3 amplitude, and parental education had no missing data. Little’s MCAR test result was non-significant (χ² (340) = 350.38, *p* = .34), suggesting that the missing data patterns were distributed completely at random (MCAR).

### 3.2 P3 amplitude reliability and intraclass correlation calculation

As shown in Table 3, zero-order correlations indicated that P3 amplitudes in both congruent and incongruent trials were strongly auto-correlated across assessments at years 12, 15, and 18. Importantly, these findings suggest that between-person differences in P3 amplitude remain relatively stable across adolescence (all *r* > .487, *p* < .001).

**Table 3:** Zero-order Pearson correlations between pairs of congruent and incongruent trial amplitudes at the three data collection times.

		**P3 - Congruent trials**			**P3 - Incongruent trials**			**CBCL/ASR**			**SCA(A)RED**			**Age**		
	**Age group**	12	15	18	12	15	18	12	15	18	12	15	18	12	15	18
**P3- Congruent trials**	12	1														
	15	.52***	1													
	18	.537***	.714***	1												
**P3 - Incongruent trials**	12	.921***	.535***	.533***	1											
	15	.491***	.946***	.691***	.555***	1										
	18	.487***	.684***	.953***	.506***	.693***	1									
	12	−.029	−.038	−.181	−.028	−.041	−.161	1								
**CBCL**^1^**/ASR**^2^	15	.074	−.038	−.054	.034	−.054	−.017	.59***	1							
	18	−.046	.029	.049	−.068	.008	.034	.024	.221*	1						
	12	−.004	−.115	−.091	−.015	−.114	−.077	.224**	.147	.1	1					
**SCA(A)RED**^3/4^	15	−.146	−.259**	−.094	−.153	−.253**	−.103	.214*	.277***	.174	.405***	1				
	18	.02	−.188	−.016	−.054	−.223*	−.011	.178*	.318***	.459***	.367***	.549***	1			
	12	.025	.159	.197	.041	.111	.175	.034	.067	−.078	.029	.071	.043	1		
**Age at EEG**	15	.073	−.047	−.041	.054	−.067	−.021	.094	−.032	−.032	.116	−.035	.012	.189	1	
	18	.092	−.034	.008	.096	−.094	−.041	−.085	−.112	−.111	.105	.105	−.071	−.093	−.065	1

*Note.* *** p < .001; ** p < .01; * p < .05; ^1^CBCL - Child Behavior Checklist; ^2^ASR - Adult Self Report; ^3^SCARED - Screen for Child Anxiety Related Emotional Disorders; ^4^SCAARED - Screen for Adult Anxiety Related Emotional Disorders.

To assess the intraclass correlations (ICC) for P3 amplitude, we constructed an empty model with a random intercept. For P3 amplitude on congruent trials, the ICC was.62, indicating that 62 % of the variability in P3 amplitude was attributable to differences between individuals, while the remaining 38 % reflected within-person variability individuals across measurements. The ICC for P3 amplitude on incongruent trials was .61.

### 3.3 Mixed-effects modeling

#### 3.3.1 Developmental processes

Age and sex were significant predictors of P3 amplitude on congruent (sex: *β* = 2.30, *t*(180.37) = 4.91, *p* < .001, *CI* 95 % [1.37, 3.22]; age: *β* = -.25, *t*(229.16) = -4.37, *p* < .001, *CI* 95 % [-.37, −.14]) and incongruent trials (sex: *β* = 2.78, *t*(177.44) = 5.49, *p* < .001, *CI* 95 % [1.78, 3.78]; age: *β* = -.34, *t*(226.58) = -5.38, *p* < .001, *CI* 95 % [-.46, −.21]). The significant age effect indicates that as age increases, P3 amplitude decreases throughout adolescence. The significant sex effect suggests that males exhibited higher P3 amplitude compared to females.

**Fig. 1:**
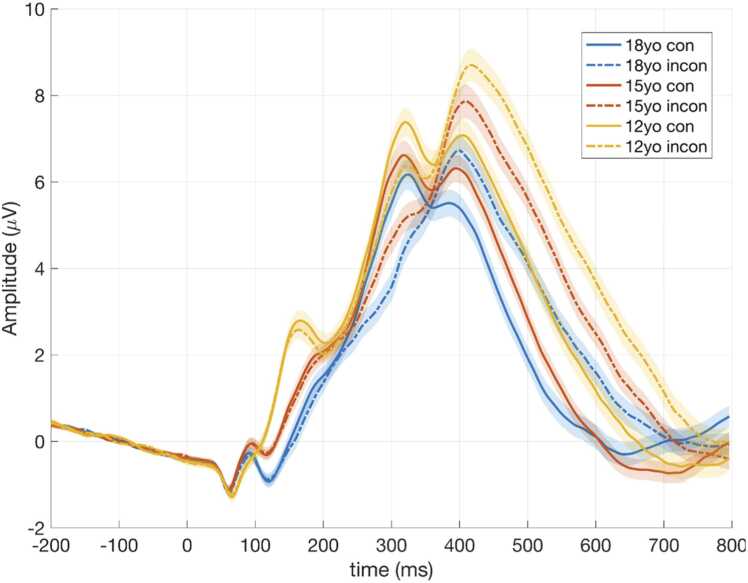
Average ERP waveforms for the electrode cluster, separated by age group and task condition. *Note.* yo - years old; ***μ***V - microvolt; con - congruent; incon - incongruent.

**Fig. 2:**
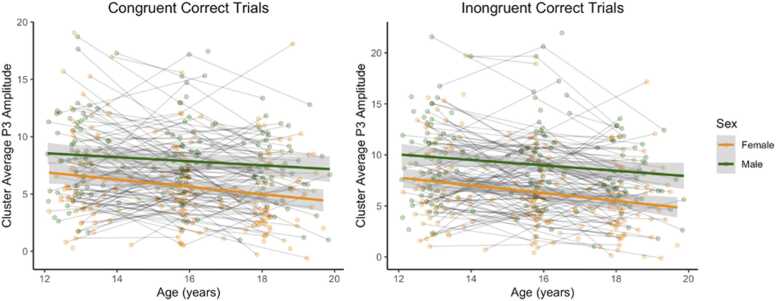
The main effect of age and sex on P3 amplitude in congruent and incongruent trials.

**Fig. 3:**
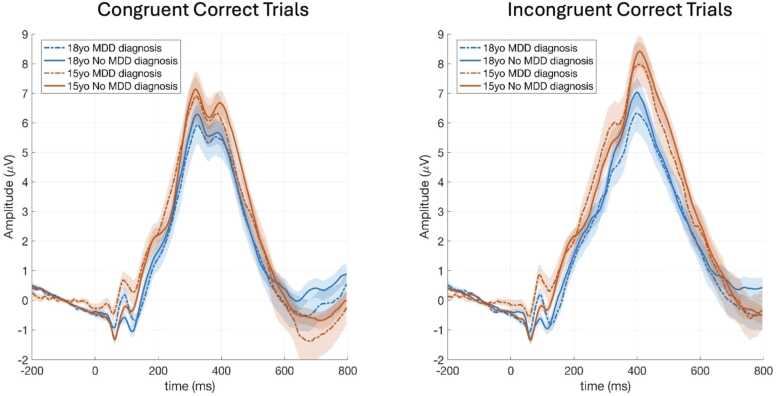
Average ERP waveforms for the electrode cluster, separated by age group, depression diagnosis, and task condition. *Note.* yo - years old; ***μ***V - microvolt; MDD - major depression diagnosis.

In a separate model, we tested the moderating effect of sex on the association between age and P3 amplitude. The interaction term (sex*age) was not statistically significant for P3 amplitude on congruent (*β* =.12, *t*(228.49) = 1.02, *p* = .31, *CI* 95 % [-.11,.34], or incongruent trials (*β* =.04, *t*(225.54) = 0.35, *p* = .73, *CI* 95 % [-.20,.29]). This suggests that males and females show similar patterns of decrease in P3 amplitude over time. See Fig. 1 for average ERP waveforms by age and task condition; Fig. 2 for associations between age and P3 amplitude by sex; and Fig. 3 for average ERP waveforms by age group, task condition, and depression diagnosis.

#### 3.3.2 Depression

*1. Continuous depression symptom score:* The within-person effect of depression – as measured by the CBCL at 12 and 15 and the ASR at age 18 – significantly predicted P3 amplitude on both congruent (*β* = −.10, *t*(63.33) = -2.16, *p* = .04, *CI* 95 % [-.19, −.01]) and incongruent trials (*β* = −.13, *t*(80.32) = -2.58, *p* = .01, *CI* 95 % [-.23, −.03]). In contrast, the between-person depression effect was not statistically significant on either the congruent (*β* = −.03, *t*(51.05) = -.33, *p* = .74, *CI* 95 % [-.20,.14]) or incongruent trials (*β* = −.05, *t*(44.04) = -.61, *p* = .55, *CI* 95 % [-.22,.12]). These results suggest that, when age- and sex-related changes in P3 amplitude are not accounted for, within-person increase in depressive symptoms predicts reduction in P3 amplitude, whereas between-person differences in depressive symptoms do not predict P3 amplitude. The RI-CLPM results, available in Section 1 of the Supplementary materials, led to similar conclusions. Briefly, the results suggested that none of the reciprocal (spill-over) associations between P3 amplitude and depression symptoms across timepoints, reached statistical significance.

*2. Depression diagnosis:* Depression as a diagnosis was not a significant predictor of P3 amplitude on congruent trials (*β* = −0.75, *t*(255.30) = -1.54, *p* = .12, *CI* 95 % [-1.71, 0.21]). However, it was a significant predictor on incongruent trials (*β* = −1.30, *t*(255.31) = -2.40, *p* = .02, *CI* 95 % [-2.37, −0.23]).

*3. Age, sex, and continuous depression symptom score:* The parameter estimates of the fixed predictors and random intercept for P3 amplitude on both congruent and incongruent trials are provided in Table 4, Table 5. As in *Model 1*, age and sex remained statistically significant. However, the significant within-person effect of depressive symptoms on reduced P3 amplitudes shown in Model 2 was no longer significant for either trial type.

**Table 4:** P3 amplitude by task condition, age group, sex, and depression diagnosis.

**Trials**	**Age**	**Sex**	**Diagnosis**	**N**	**Mean P3 (µV)**	**Min P3**	**Max P3**	**SD P3**
**Congruent**	**12 year**	Female	No MDD	55	6.789	0.303	16.981	3.987
			Diagnosis missing	13	6.738	2.491	19.047	4.537
		Male	No MDD	47	8.352	1.654	18.724	3.582
			MDD	1	4.912	4.912	4.912	NA
			Diagnosis missing	17	8.516	2.649	17.292	3.883
	**15 year**	Female	No MDD	44	5.206	0.501	15.578	2.958
			MDD	12	6.641	2.779	12.053	3.061
			Diagnosis missing	19	4.74	0.627	11.764	2.655
		Male	No MDD	47	8.441	1.86	17.448	3.835
			MDD	5	7.005	4.906	10.339	2.165
			Diagnosis missing	10	6.402	0.99	11.014	2.768
	**18 year**	Female	No MDD	31	4.475	−0.593	10.968	2.896
			MDD	22	5.135	1.343	11.582	3.117
			Diagnosis missing	11	5.781	−0.283	18.098	5.234
		Male	No MDD	32	7.334	2.999	13.358	2.447
			MDD	9	7.423	0.993	12.79	4.182
			Diagnosis missing	3	6.801	3.105	9.721	3.375
**Incongruent**	**12 year**	Female	No MDD	55	7.716	0.698	19.717	4.242
			Diagnosis missing	13	6.901	2.908	15.611	3.62
			No MDD	47	9.7	2.346	21.578	3.899
			MDD	1	8.267	8.267	8.267	NA
			Diagnosis missing	17	9.599	2.686	19.666	4.377
	**15 year**	Female	No MDD	44	5.791	0.905	18.927	3.372
			MDD	12	7.355	3.346	11.792	2.875
			Diagnosis missing	19	5.297	0.412	11.241	2.546
		Male	No MDD	47	9.919	2.002	21.946	4.577
			MDD	5	8.478	6.208	10.474	1.782
			Diagnosis missing	10	7.352	1.027	15.858	3.886
	**18 year**	Female	No MDD	31	5.464	−0.116	12.079	3.16
			MDD	22	5.402	1.149	11.78	3.177
			Diagnosis missing	11	5.678	0.032	17.162	5.028
		Male	No MDD	32	8.025	2.873	13.561	2.776
			MDD	9	7.75	1.802	12.435	3.731
			Diagnosis missing	3	7.626	4.659	11.777	3.704

*Note.* Diagnostic group is based on binary KSADS/SCID assessment (No MDD = did not meet diagnostic criteria for a major depressive disorder diagnosis, MDD = met diagnostic criteria for major depression diagnosis; Missing data = did not complete the KSADS/SCID).

**Table 5:** Fixed and Random Effects Parameter Estimates for Congruent and Incongruent Correct Trials in the Mixed-Effects Model.

**Variables**	**Congruent trials**					**Incongruent trials**				
***Fixed Effects***	**Estimate (SE)**	**t-score (DF)**	**p-value**	**CI 95 % (Lower)**	**CI 95 % (Upper)**	**Estimate (SE)**	**t-score (DF)**	**p-value**	**CI 95 % (Lower)**	**CI 95 % (Upper)**
**Intercept**	5.612 (.32)	17.742 (173.976)	< .001	4.988	6.236	6.272 (.35)	18.164 (169.91)	< .001	5.59	6.954
**Depression, within-person**	−.009 (.05)	−.169 (184.17)	.866	−.110	.092	−.01 (0.06)	−.186 (179.83)	.853	−.120	.099
**Depression, between-person**	.02 (.08)	.252 (189.47)	.801	−.136	.175	.011 (.09)	.133 (185.23)	.894	−.158	.181
**Sex (Male = 1)**	2.272 (.48)	4.721 (172.13)	< .001	1.322	3.221	2.789 (.53)	5.309 (168.11)	< .001	1.752	3.826
**Age in years**	−.229 (.07)	−3.234 (226.53)	.001	−.369	−.089	−.304 (.08)	−3.959 (221.92)	< .001	−0.456	−.153

***Random Effects***	**Estimate (SE)**	**z**	**p-value**	**CI 95 % (Lower)**	**CI 95 % (Upper)**	**Estimate (SE)**	**z**	**p-value**	**CI 95 % (Lower)**	**CI 95 % (Upper)**
**Intercept**	7.52 (2.74)	2.743	.006	2.146	12.894	9.01 (3)	3.001	.003	3.126	14.894

*4. Age, sex, and depression diagnosis:* In line with *Model 3 A*, age and sex were significant predictors of P3 amplitude in both congruent and incongruent trials. However, having a depression diagnosis was not a significant predictor of P3 amplitude on either congruent (*β* =.20, *t*(269.33) = .38, *p* = .70, *CI* 95 % [-.81, 1.20]) or incongruent trials (*β* = −.09, *t*(269.14) = -.15, *p* = .88, *CI* 95 % [-1.19, 1.02]). These results suggest that when age and sex are co-varied with depression, depression is no longer a significant predictor of P3 amplitude in either trial type. See Table 4 for data on P3 amplitude by task condition, age group, sex, and depression diagnosis.

### 3.4 Anxiety

*1. Anxiety symptom score:* The within-person effect of anxiety – as measured by the SCARED at 12 and 15 and the SCAARED at age 18 – did not significantly predict P3 amplitude on both congruent (*β* = −.02, *t*(154.12) = -1.25, *p* = .21, *CI* 95 % [-0.06, 0.01]) and incongruent trials (*β* = −.02, *t*(148.80) = -1.02, *p* = .31, *CI* 95 % [-.06,.02]). Furthermore, the between-person anxiety effect was not statistically significant on either the congruent (*β* = −.04, *t*(175.66) = -1.63, *p* = .10, *CI* 95 % [-.08,.01]) and incongruent trials (*β* = −0.04, *t*(170.08) = -1.73, *p* = .08, *CI* 95 % [-0.09, 0.01]). These results suggest that within- and between-person changes in anxiety symptoms do not predict change in P3 amplitude, even when age- and sex-related changes in P3 amplitude are not accounted for.

*2. Age, sex, and anxiety symptoms score:* Age and sex were statistically significant predictors in both congruent (sex: *β* = 2.47, *t*(171.03) = 4.84, *p* < .001, *CI* 95 % [1.46, 3.48]; age: *β* = -.23, *t*(204.04) = -3.67, *p* < .001, *CI* 95 % [-.35, −.10]) and incongruent trials (sex: *β* = 3.00, *t*(164.86) = 5.46, *p* < .001, *CI* 95 % [1.92, 4.09]; age: *β* = -.30, *t*(200.12) = -4.44, *p* < .001, *CI* 95 % [-.43, −.17]). With age and sex included in the model, the between- and within-person effects of anxiety were not significant predictors of P3 amplitude in either congruent (within-person: *β* = −.01, *t*(157.82) = -.52, *p* = .60, *CI* 95 % [-.04,.02]; between-person: *β* = .01, *t*(180.62) = .45, *p* = .65, *CI* 95 % [-.03,.05]) or incongruent trials (within-person: *β* =.00, *t*(152.24) = -.16, *p* = .87, *CI* 95 % [-.04,.03]; between-person: *β* = .01, *t*(174.85) = .60, *p* = .55, *CI* 95 % [-.03,.06]). In sum, these results suggest that increases in between- and within-person anxiety severity are not associated with change in P3 amplitude in either trial type, while age and sex remain significant.

### 3.5 Secondary analyses with peak P3 amplitude

The results were consistent with those examining the average P3 amplitude component across sites. Specifically, within-person depression scores significantly predicted decreases in P3 peak amplitude when age and sex were not included as covariates. However, this association diminished to non-significance once age and sex were added to the model, while age and sex remained significant predictors. For anxiety symptoms, neither between- nor within-person SCA(A)RED scores predicted P3 peak amplitude, regardless of whether age and sex were included. In contrast, age and sex consistently emerged as significant predictors of P3 peak amplitude across models. Finally, depression diagnosis (KSADS/SCID) was not a significant between-person predictor of P3 peak amplitude, with or without covariates, but again, age and sex remained significant when included. Overall, these findings further support the robustness of the age and sex effects observed in our primary analyses and suggest that focusing on the maximal P3 amplitude site yields similar conclusions.

## 4 Discussion

The present longitudinal study builds on prior work that has examined either cross-sectional or prospective associations based on single assessments of P3 amplitude and depressive symptoms (Greimel et al., 2015, Houston et al., 2003, 2004; Santopetro et al., 2020, Santopetro et al., 2022, 2023), by investigating their dynamic association using repeated measures of both constructs across adolescence. Unlike these prior reports, however, our study includes repeated assessments of both P3 amplitude and depressive symptoms. Within a neurodevelopmental framework, we delineated longitudinal associations of P3 amplitude with age, sex, and depressive symptoms in a cohort of adolescents assessed at 12, 15, and 18 years of age, at increased early temperamental risk for emotional disorders. Critically, the multi-wave data enabled specification of within- and between-person effects on P3 amplitude. The results demonstrated that within-person P3 amplitude is highly reliable, even across EEG assessments more than 6 years apart, making it an ideal measure for studying individual differences in developmental processes and vulnerability factors. Furthermore, variability in P3 amplitude across adolescence was most strongly predicted by age, or age-related decline in P3 amplitude over time, as well as sex differences that were consistent across time. In contrast, although within-person increases in depressive symptoms across adolescence similarly appeared to predict decreases in P3 amplitude, these effects were diminished to nonsignificance once age and sex were added to the models. This suggests that the variance in P3 amplitude associated with depressive symptoms may substantially overlap with age-related change. In other words, depression and age are highly intertwined across adolescence, making it difficult to separate unique contributions of both in adolescent cohorts. However, to the extent possible given the nature of the data we collected, our models demonstrated that age remained a significant predictor of P3 amplitude even when controlling for depressive symptoms, whereas the depression effect was reduced to non-significance. Again, to the extent possible with our longitudinal data, this pattern indicates that age-related decline in P3 amplitude may be the more robust driver of variability in P3 amplitude change during adolescence. These results underscore the value of longitudinal designs in studying neurodevelopmental disorders like depression, as cross-sectional studies cannot distinguish between depression-specific effects and normative age-related changes in P3 amplitude. At the same time, our findings do not rule out depression-related influences entirely, but rather highlight the complexity of parsing their effects from developmental trajectories.

Consistent with previous cross-sectional studies, we found a significant longitudinal association between within-person depressive symptoms and reduced P3 amplitude when we did not account for the variability explained by age and sex. In the supplementary materials we also showed that when P3 amplitude is the independent variable and depression is the dependent variable, within-person P3 amplitude significantly predicts depression on incongruent trials (p = .01) and the association is marginally significant on congruent trials (p = .099). Considering that across all analyses, the associations became non-significant when age and sex were included in the model, we show that both a *'biomarker'*-type model—in which symptoms predict a neural marker—and a symptom-focused model—in which a biomarker predicts a symptom—similarly produce null effects. Taken together, our findings highlight a key limitation of cross-sectional designs: overlapping variance in age and depression effects on P3 amplitude makes it difficult to disentangle unique psychopathological influences from developmental effects – with developmental factors emerging as more robust in our longitudinal models. Regarding age and sex, our findings in adolescents are consistent with previous research in adults showing that with increasing age, P3 amplitude decreases (Kangas et al., 2022). The relatively early onset of P3 amplitude reduction shown here may be related to the choice of the study sample, which is at higher risk for developing internalizing symptoms than a community sample would be, potentially associated with an earlier P3 amplitude decline. Sex added further nuance to this association, with males exhibiting higher P3 amplitudes than females. Given that depressive symptoms are higher in adolescent girls than boys, and that P3 amplitude is lower in girls, as we demonstrate here, sex differences could confound the effects of depression on P3 amplitude. What may appear to be a depression effect could reflect a sex and age effect, or at least highlight the interrelationship between these factors. These complexities need to be addressed in a comprehensive model that accounts for both developmental processes and depression-related changes in brain outcomes. Understanding the interplay of these factors is essential for identifying potential neurobiological markers of depression risk.

Additionally, we explored the association between anxiety and P3 amplitude to determine whether changes in P3 amplitude are differentially related to changes in depressive symptoms or if P3 amplitude functions as a transdiagnostic marker of neurobiological vulnerability to internalizing disorders. Our results indicate that within- and between-person effects of anxiety are not significant predictors of P3 amplitude.

There are several strengths and limitations to this study that should be considered when interpreting the findings. One limitation is the measurement inconsistency over time in our continuous depression measure, as we switched from the child version (CBCL) to the adult version (ASR) of the questionnaire. Additionally, there was informant inconsistency, with parents completing the CBCL at years 12 and 15, and participants completing the measure themselves at year 18. Whereas parent report is commonly used in childhood and early adolescence, we acknowledge that for internalizing symptoms like depression, self-report is often considered more accurate, as adolescents may have greater insight into their internal emotional experiences than their parents. Relying on parent-reported data may have led to underestimation of depressive symptoms or confounding by parental characteristics, including parents’ own mental health. These measurement and informant inconsistencies may introduce bias, potentially affecting the reliability and validity of the results. Furthermore, the sample was primarily recruited based on early temperamental risk for anxiety rather than depression. As a result, while there is high comorbidity between anxiety and depression, the generalizability of our findings to the general population may be limited due to biases in observed associations introduced by non-random sampling at baseline. Furthermore, although the sample was enriched for internalizing symptom risk, it is not representative of a clinically depressed population, and the results may differ in such a population. Consistent with this, the proportion of participants with a depression diagnosis was very low at age 12 (N = 1), followed by a developmentally expected increase over time (age 15, N = 17; age 18, N = 30), which suggests limited power to detect associations with clinical depression at earlier ages. Future research should examine similar questions in youth oversampled for early vulnerability to depression, such as those with a family history of the disorder or genetic risk factors. Given that P3 amplitude has a known genetic component (Baal et al., 1998, Wright et al., 2001), studies that explore the relationship between depression and P3 amplitude in genetically at-risk populations could contribute to the growing field of endophenotype research, potentially identifying neurobiological indicators of depression vulnerability. It is also important to note that the stimulus-locked P3 amplitude on the Flanker task may overlap with the motor response as well as the response-related ERPs, including response- and error-related components. However, our analyses focused on correct trials and modeled congruent and incongruent trials separately, which reduces the potential confounding effect of errors. Nevertheless, some residual overlap may remain. The advantage of this measure is that it has been used in multiple prior studies examining issues related to the focus in the current paper (Santopetro et al., 2020, Santopetro et al., 2021, 2023). Despite these limitations, the study has important strengths. The longitudinal nature of our dataset, which spans a wide age range from 12 to 18 years, enables nuanced understanding of how P3 amplitude changes across adolescence.

In conclusion, our findings evidence a strong neurodevelopmental trajectory of P3 amplitude across adolescence, as well as significant effects of sex during this period. Thus, whereas depressive symptoms have been associated with reduced P3 amplitude in prior cross-sectional studies, our longitudinal analyses suggest that age effects likely account for shared variance and attenuate the unique contribution of depressive symptoms. Thus, age emerged as the more robust predictor of P3 amplitude when modeled alongside depression across time. These results underscore the need for future studies that account for developmental changes in both brain responses and symptoms of psychopathology. Future research should seek to replicate these findings in larger samples, including those oversampled for vulnerability to depression.

## CRediT authorship contribution statement

**Daniel S. Pine:** Writing – review & editing, Supervision, Resources, Methodology, Investigation, Conceptualization. **Lucrezia Liuzzi:** Writing – review & editing, Supervision, Software, Methodology, Formal analysis, Data curation, Conceptualization. **Selin Zeytinoglu:** Writing – review & editing, Supervision, Project administration, Methodology, Investigation, Data curation, Conceptualization. **Katharina Kircanski:** Writing – review & editing, Writing – original draft, Supervision, Resources, Methodology, Investigation, Formal analysis, Data curation, Conceptualization. **Nathan A. Fox:** Writing – review & editing, Supervision, Software, Resources, Project administration, Methodology, Investigation, Funding acquisition, Conceptualization. **Marco McSweeney:** Writing – review & editing, Supervision, Project administration, Methodology, Formal analysis, Data curation, Conceptualization. **Marta Korom:** Writing – review & editing, Writing – original draft, Visualization, Software, Methodology, Formal analysis, Data curation, Conceptualization.

## Funding

This work was supported by the National Institute of Mental Health Intramural Research Program project ZIA-MH002782 and the National Institute of Health grants MH093349 and HD017899 awarded to N.A.F.

## Declaration of Competing Interest

The authors declare that they have no known competing financial interests or personal relationships that could have appeared to influence the work reported in this paper.

## Data Availability

Data will be made available on request.

## References

[bib1] Achenbach T.M. (2000).

[bib2] Achenbach T.M. (2011).

[bib3] Achenbach T.M., Rescorla L.A. (2014).

[bib4] Andersen S.L., Teicher M.H. (2008). Stress, sensitive periods and maturational events in adolescent depression. Trends Neurosci..

[bib5] Angulo M., Rooks B.T., Gill M., Goldstein T., Sakolsky D., Goldstein B., Birmaher B. (2017). Psychometrics of the screen for adult anxiety related disorders (SCAARED)- A new scale for the assessment of DSM-5 anxiety disorders. Psychiatry Res..

[bib6] Ashford J.W., Coburn K.L., Rose T.L., Bayley P.J. (2011). P300 energy loss in aging and alzheimer’s disease. J. Alzheimer’S. Dis..

[bib7] Baal G.C.M. van, Geus E.J.C. de, Boomsma D.I. (1998). Longitudinal study of genetic influences on ERP-P3 during childhood. Dev. Neuropsychol..

[bib8] Barker T.V., Buzzell G.A., Troller-Renfree S.V., Bowman L.C., Pine D.S., Fox N.A. (2021). The influence of social motivation on neural correlates of cognitive control in girls. Dev. Psychobiol..

[bib9] Beaujean, A.A. (2012). BaylorEdPsych: R package for Baylor University educational psychology quantitative courses. Retrieved from 〈https://CRAN.R-project.org/package=BaylorEdPsych〉.

[bib10] Birmaher B., Khetarpal S., Brent D., Cully M., Balach L., Kaufman J., Neer S.M. (1997). The Screen for Child Anxiety Related Emotional Disorders (SCARED): Scale construction and psychometric characteristics. J. Am. Acad. Child Adolesc. Psychiatry.

[bib11] Brennan G.M., Baskin-Sommers A.R. (2018). Brain-behavior relationships in externalizing: P3 amplitude reduction reflects deficient inhibitory control. Behavioural Brain Research.

[bib12] Bruder G.E., Kroppmann C.J., Kayser J., Stewart J.W., McGrath P.J., Tenke C.E. (2009). Reduced brain responses to novel sounds in depression: P3 findings in a novelty oddball task. Psychiatry Res..

[bib13] Buzzell G.A., Barker T.V., Troller-Renfree S.V., Bernat E.M., Bowers M.E., Morales S., Fox N.A. (2019). Adolescent cognitive control, theta oscillations, and social observation. NeuroImage.

[bib14] Buzzell G.A., Troller-Renfree S.V., Barker T.V., Bowman L.C., Chronis-Tuscano A., Henderson H.A., Fox N.A. (2017). A neurobehavioral mechanism linking behaviorally inhibited temperament and later adolescent social anxiety. J. Am. Acad. Child Adolesc. Psychiatry.

[bib15] Calkins S.D., Fox N.A., Marshall T.R. (1996). Behavioral and physiological antecedents of inhibited and uninhibited behavior. Child Dev..

[bib16] Clayson P.E., Baldwin S.A., Larson M.J. (2012). How does noise affect amplitude and latency measurement of event-related potentials (ERPs)? A methodological critique and simulation study. Psychophysiology.

[bib17] Conte S., Richards J.E., Fox N.A., Valadez E.A., McSweeney M., Tan E., Pine D.S., Winkler A.M., Liuzzi L., Cardinale E.M., White L.K., Buzzell G.A. (2023). Multimodal study of the neural sources of error monitoring in adolescents and adults. Psychophysiology.

[bib18] Curran P.J., Bauer D.J. (2011). The disaggregation of within-person and between-person effects in longitudinal models of change. Annu. Rev. Psychol..

[bib19] Debener S., Thorne J., Schneider T.R., Viola F.C. (2010).

[bib20] Debnath R., Buzzell G.A., Morales S., Bowers M.E., Leach S.C., Fox N.A. (2020). The Maryland analysis of developmental EEG (MADE) pipeline. Psychophysiology.

[bib21] Delorme A., Makeig S. (2004). EEGLAB: an open source toolbox for analysis of single-trial EEG dynamics including independent component analysis. J. Neurosci. Methods.

[bib22] Dinteren R. van, Arns M., Jongsma M.L.A., Kessels R.P.C. (2014). P300 Development across the Lifespan: a systematic review and meta-analysis. PLoS ONE.

[bib23] Donchin E. (1981). Surprise!… Surprise?. Psychophysiology.

[bib24] Ehlers C.L., Wall T.L., Garcia-Andrade C., Phillips E. (2001). Auditory P3 findings in mission Indian youth. J. Stud. Alcohol.

[bib25] Enders C.K. (2022).

[bib26] Eriksen C.W. (1995). The flankers task and response competition: A useful tool for investigating a variety of cognitive problems. Vis. Cogn..

[bib27] Eriksen B.A., Eriksen Ch.W. (1974). Effects of noise letters upon the identification of a target letter in a nonsearch task. Percept. Psychophys..

[bib28] Faja S., Clarkson T., Webb S.J. (2016). Neural and behavioral suppression of interfering flankers by children with and without autism spectrum disorder. Neuropsychologia.

[bib29] Feldmann L., Piechaczek C.E., Pehl V., Bartling J., Bakos S., Schulte-Körne G., Greimel E. (2018). State or trait? Auditory event-related potentials in adolescents with current and remitted major depression. Neuropsychologia.

[bib30] First M.B. (2015). Structured Clinical Interview for the *DSM* ( SCID). Encycl. Clin. Psychol..

[bib31] Fuhrmann D., Knoll L.J., Blakemore S.-J. (2015). Adolescence as a sensitive period of brain development. Trends Cogn. Sci..

[bib32] Greimel E., Trinkl M., Bartling J., Bakos S., Grossheinrich N., Schulte-Körne G. (2015). Auditory selective attention in adolescents with major depression: An event-related potential study. J. Affect. Disord..

[bib33] Gustavson K., von Soest T., Karevold E. (2012). Attrition and generalizability in longitudinal studies: findings from a 15-year population-based study and a Monte Carlo simulation study. BMC Public Health.

[bib34] Hoffman L., Stawski R.S. (2009). Persons as contexts: Evaluating between-person and within-person effects in longitudinal analysis. Res. Hum. Dev..

[bib35] Houston R.J., Bauer L.O., Hesselbrock V.M. (2003). Depression and familial risk for substance dependence:a P300 study of young women. Psychiatry Res. Neuroimaging.

[bib36] Johnstone S.J., Galletta D. (2013). Event-rate effects in the flanker task: ERPs and task performance in children with and without AD/HD. Int. J. Psychophysiol. Off. J. Int. Organ. Psychophysiol..

[bib37] Kalin N.H. (2020). The critical relationship between anxiety and depression. Am. J. Psychiatry.

[bib38] Kangas E.S., Vuoriainen E., Lindeman S., Astikainen P. (2022). Auditory event-related potentials in separating patients with depressive disorders and non-depressed controls: a narrative review. Int. J. Psychophysiol..

[bib39] Kaufman J., Birmaher B., Brent D., Rao U., Flynn C., Moreci P., Ryan N. (1997). Schedule for affective disorders and schizophrenia for school-age children-present and lifetime version. Am. Psychol. Assoc..

[bib40] Klawohn J., Joyner K., Santopetro N., Brush C.J., Hajcak G. (2022). Depression reduces neural correlates of reward salience with increasing effort over the course of the progressive ratio task. J. Affect. Disord..

[bib41] Kuznetsova A., Brockhoff P.B., Christensen R.H.B. (2017). lmerTest package: Tests in linear mixed effects models.

[bib42] Launes J., Hokkanen L., Laasonen M., Tuulio-Henriksson A., Virta M., Lipsanen J., Tienari P.J., Michelsson K. (2014). Attrition in a 30-year follow-up of a perinatal birth risk cohort: factors change with age. PeerJ.

[bib43] Leach S.C., Morales S., Bowers M.E., Buzzell G.A., Debnath R., Beall D., Fox N.A. (2020). Adjusting ADJUST: optimizing the ADJUST algorithm for pediatric data using geodesic nets. Psychophysiology.

[bib44] Lepistö T., Soininen M., Čeponien≐ R., Almqvist F., Näätänen R., Aronen E.T. (2004). Auditory event-related potential indices of increased distractibility in children with major depression. Clin. Neurophysiol..

[bib45] Little R.J.A. (1988). A test of missing completely at random for multivariate data with missing values. J. Am. Stat. Assoc..

[bib46] Liuzzi L., Pine D.S., Fox N.A., Averbeck B.B. (2023). Changes in behavior and neural dynamics across adolescent development. J. Neurosci..

[bib47] McSweeney M., Morales S., Valadez E.A., Buzzell G.A., Fox N.A. (2021). Longitudinal age- and sex-related change in background aperiodic activity during early adolescence. Dev. Cogn. Neurosci..

[bib48] Melynyte S., Wang G.Y., Griskova-Bulanova I. (2018). Gender effects on auditory P300: A systematic review. Int. J. Psychophysiol..

[bib49] Mognon A., Jovicich J., Bruzzone L., Buiatti M. (2011). ADJUST: An automatic EEG artifact detector based on the joint use of spatial and temporal features. Psychophysiology.

[bib50] Morales S., Zeytinoglu S., Buzzell G.A., Valadez E.A., Troller-Renfree S.V., Bowers M.E., Chronis-Tuscano A., Degnan K.A., Almas A.N., Pine D.S., Fox N.A. (2022). Neurocognitive profiles in adolescence predict subsequent anxiety trajectories during the COVID-19 pandemic. Biolog. Psychiatry Cognitive Neurosci. Neuroim..

[bib51] Nolan H., Whelan R., Reilly R.B. (2010). FASTER: fully automated statistical thresholding for EEG artifact rejection. J. Neurosci. Methods.

[bib52] Perrin F., Pernier J., Bertrand O., Echallier J.F. (1989). Spherical splines for scalp potential and current density mapping. Electroencephalogr. Clin. Neurophysiol..

[bib53] Pfueller U., Oelkers-Ax R., Gmehlin D., Parzer P., Roesch-Ely D., Weisbrod M., Bender S. (2011). Maturation of P300 amplitude and short-term learning as reflected by P300 habituation between trial blocks in children. Int. J. Psychophysiol..

[bib54] Polich J. (2007). Updating P300: an integrative theory of P3a and P3b. Clin. Neurophysiol..

[bib55] Polich J., Ladish C., Burns T. (1990). Normal variation of P300 in children: Age, memory span, and head size. Int. J. Psychophysiol..

[bib56] Riggins T., Scott L.S. (2019). P300 development from infancy to adolescence. Psychophysiology.

[bib57] Röschke J., Wagner P. (2003). A confirmatory study on the mechanisms behind reduced P300 waves in depression. Neuropsychopharmacology.

[bib58] Rossini P.M., Rossi S., Babiloni C., Polich J. (2007). Clinical neurophysiology of aging brain: from normal aging to neurodegeneration. Prog. Neurobiol..

[bib59] Saliasi E., Geerligs L., Lorist M.M., Maurits N.M. (2013). The relationship between P3 amplitude and working memory performance differs in young and older adults. PloS one.

[bib60] Salk R.H., Hyde J.S., Abramson L.Y. (2017). Gender differences in depression in representative national samples: meta-analyses of diagnoses and symptoms. Psychol. Bull..

[bib61] Santopetro N.J., Brush C.J., Bruchnak A., Klawohn J., Hajcak G. (2021). A reduced P300 prospectively predicts increased depressive severity in adults with clinical depression. Psychophysiology.

[bib62] Santopetro N.J., Kallen A.M., Threadgill A.H., Hajcak G. (2020). Reduced flanker P300 prospectively predicts increases in depression in female adolescents. Biol. Psychol..

[bib63] Santopetro N.J., Kallen A.M., Threadgill A.H., Amir N., Hajcak G. (2021). Blunted flanker P300 demonstrates specificity to depressive symptoms in females during adolescence. Res. Child Adolesc. Psychopathol..

[bib64] Santopetro N.J., Mulligan E.M., Brush C.J., Hajcak G. (2022). Reduced P300 amplitude is consistently associated with trait anhedonia across repeated assessments. Psychophysiology.

[bib65] Shner-Livne G., Buzzell G.A., Fox N.A., Shechner T. (2024). Induced error-related theta activity, not error-related negativity, predicts task performance as well as anxiety and worry during real-life stress in a youth sample. Psychophysiology.

[bib66] Szuromi B., Czobor P., Komlósi S., Bitter I. (2010). P300 deficits in adults with attention deficit hyperactivity disorder: a meta-analysis. Psychol. Med..

[bib67] Teixeira R., Queiroga A.C., Freitas A.I., Lorthe E., Santos A.C., Moreira C., Barros H. (2021). Completeness of retention data and determinants of attrition in birth cohorts of very preterm infants: a systematic review. Front. Pediatr..

[bib68] Thapar A., Collishaw S., Pine D.S., Thapar A.K. (2012). Depression in adolescence. Lancet.

[bib69] Thompson B., Santopetro N., Albanese B., Schmidt N.B. (2025). Depression-specific P300 deficits compared to other forms of internalizing psychopathology. J. Affect. Disord. Rep..

[bib70] Tsai M.L., Hung K.L., Lu H.H. (2012). Auditory event-related potentials in children with attention deficit hyperactivity disorder. Pediatr. Neonatol..

[bib71] Tsai M.L., Hung K.L., Tao-Hsin Tung W., Chiang T.R. (2012). Age-changed normative auditory event-related potential value in children in Taiwan. J. Formos. Med. Assoc..

[bib72] Vuorre, M., & Bolger, N. (2018). Within-subject mediation analysis for experimental data in cognitive psychology and neuroscience. 10.3758/s13428-017-0980-9. 29247385

[bib73] Walhovd K.B., Rosquist H., Fjell A.M. (2008). P300 amplitude age reductions are not caused by latency jitter. Psychophysiology.

[bib74] Wright M.J., Hansell N.K., Geffen G.M., Geffen L.B., Smith G.A., Martin N.G. (2001). Genetic influence on the variance in P3 amplitude and latency. Behav. Genet..

