# Supplementary material for: Age and sex, but not depression or anxiety, predict P3 amplitude during adolescence
Source: Dev Cogn Neurosci. 2025 Nov 1;76:101640. doi: 10.1016/j.dcn.2025.101640 (PMC12640051; doi:10.1016/j.dcn.2025.101640)
Supplement: Supplementary file 1 — Supplementary material [file mmc1.docx]

**Supplementary Materials**

**Section 1**

Random intercept cross-lagged panel models (RI-CLPM) were conducted to examine the bidirectional association between P3 amplitude, depression, and anxiety symptoms measured at years 12, 15, and 18. The models demonstrated excellent fit (see Supplementary Table 1.)

Supplementary Table 1

*Comparison of Model Fit Indices and Goodness-of-Fit Criteria for the P3-Depression and the P3-Anxiety models*

| **Fit Index** | **Goodness-of-Fit Criteria** | **P3-Depression model** | **P3 -Anxiety model** |
| --- | --- | --- | --- |
| Chi-square Test | *p* > .5 | (χ² = 0.476, *df* = 1, *p* = .49) | (χ² = 0.226, *df* = 1, *p* = .635) |
| CFI^1^ | > .95 | 1 | 1.046 |
| TLI^2^ | > .95 | 1 | 1.068 |
| RMSEA^3^ | < 0.06 | .000 | .000 |
| SRMR^4^ | < 0.08 | .014 | .006 |

**Note.** ^1^CFI - Comparative Fit Index; ^2^TLI - Tucker-Lewis Index; ^3^RMSEA - The Root Mean Square Error of Approximation; ^4^SRMR - Standardized Root Mean Square Residual

**Model results**

*P3-Depression model*

The model tested both within- and between-person effects over time. The results suggest that none of the spill-over effects, which would indicate reciprocal influences between P3 amplitude and depression symptoms across timepoints, reached statistical significance. See *Supplementary Table 2* for detailed model results.

*P3-Anxiety model*

See Supplementary Table 3 for detailed model results. None of the spill-over effects reached statistical significance.

Supplementary Table 2

*Random Intercept Cross-Lagged Panel Model Results for the P3-Depression Model.*

| **Effects** | **Parameters** | **Estimate** | **SE** | **z** | **p-value** |
| --- | --- | --- | --- | --- | --- |
| **Between-level** | |  |  |  |  |
| Random intercepts | P3 amplitude ↔ Depression | 5.536 | 13.513 | .41 | .682 |
| **Within-level**  (residual components) |  |  |  |  |  |
| Correlations | P3 amplitude, year 12 ↔ Depression, year 12 | -5.937 | 13.531 | -.439 | .661 |
|  | P3 amplitude, year 15 ↔ Depression, year 15 | .504 | 1.26 | .40 | .689 |
|  | P3 amplitude, year 18 ↔ Depression, year 18 | .092 | 1.317 | .07 | .944 |
| Carry-over effects | P3 amplitude, year 12 → P3 amplitude, year 15 | -.626 | .261 | -2.397 | .017 |
|  | P3 amplitude, year 15 → P3 amplitude, year 18 | 1.116 | .275 | 4.064 | <.001 |
|  | Depression, year 12 → Depression, year 15 | 1.185 | .314 | 3.777 | <.001 |
|  | Depression, year 15 → Depression, year 18 | .743 | .206 | 3.6 | <.001 |
| Spill-over effects | P3 amplitude, year 12 → Depression, year 15 | .162 | .188 | .859 | .39 |
|  | P3 amplitude, year 15 → Depression, year 18 | -.053 | .326 | -.163 | .871 |
|  | Depression, year 12 → P3 amplitude, year 15 | -2.205 | 2.434 | -.906 | .365 |
|  | Depression, year 15 → P3 amplitude, year 18 | .006 | .132 | .049 | .961 |

Supplementary Table 3

*Random Intercept Cross-Lagged Panel Model Results for the P3-Anxiety Model.*

| **Effects** | **Parameters** | **Estimate** | **SE** | **z** | **p-value** |
| --- | --- | --- | --- | --- | --- |
| **Between-level** | |  |  |  |  |
| Random intercepts | P3 amplitude ↔ Anxiety | -2.257 | 4.273 | -0.528 | .597 |
| **Within-level**  (residual components) |  |  |  |  |  |
| Correlations | P3 amplitude, year 12 ↔ Anxiety, year 12 | 0.704 | 5.299 | 0.133 | .894 |
|  | P3 amplitude, year 15 ↔ Anxiety, year 15 | -9.596 | 6.02 | -1.594 | .111 |
|  | P3 amplitude, year 18 ↔ Anxiety, year 18 | -2.423 | 4.09 | -0.592 | .554 |
| Carry-over effects | P3 amplitude, year 12 → P3 amplitude, year 15 | -0.592 | 0.193 | -3.065 | .002 |
|  | P3 amplitude, year 15 → P3 amplitude, year 18 | 0.703 | 0.315 | 2.233 | .026 |
|  | Anxiety, year 12 → Anxiety, year 15 | 0.232 | 0.249 | 0.932 | .351 |
|  | Anxiety, year 15 → Anxiety, year 18 | 0.376 | 0.22 | 1.709 | .087 |
| Spill-over effects | P3 amplitude, year 12 → Anxiety, year 15 | -0.905 | 0.94 | -0.963 | .335 |
|  | P3 amplitude, year 15 → Anxiety, year 18 | -1.588 | 1.123 | -1.413 | .158 |
|  | Anxiety, year 12 → P3 amplitude, year 15 | -0.024 | 0.093 | -0.257 | .797 |
|  | Anxiety, year 15 → P3 amplitude, year 18 | 0.007 | 0.028 | 0.246 | .806 |

**Note.** Anxiety scores represent SCA(A)RED scores.

**Section 2**

The mixed-effects models were rerun with the continuous depression and anxiety measures as the outcome variables and the within- and between-person P3 amplitude as predictors, both with and without age and sex as covariates.

***Developmental processes***

We progressively increased the complexity of the models and examined fit indices to identify the model that best captured the relationship between age and depression. All models included a random intercept. The first model included age as a linear fixed slope. The second model treated age as a linear random slope. The third model included age as both a random linear and nonlinear (quadratic) slope. The third model fit the data best (first model: *AIC* = 1,943.87, *BIC* = 1,963.33, *Log-likelihood* = -966.94; second model: *AIC* = 1,934.36, *BIC* = 1,957.71, *Log-likelihood* = -961.18; first vs. second model comparison: *χ2*(1) = 11.51, *p* <.001; third model: *AIC* = 1,802.67, *BIC* = 1,845.48, *Log-likelihood* = -890.34; second vs. third model comparison: *χ2*(5) = 141.69, *p* <.001).

The linear and quadratic age terms were significant predictors of depression (linear age: *β* = .89, *t*(115.76) = 9.07, *p* < .001, *CI* 95% [.70, 1.09]; quadratic age: *β* = .21, *t*(103.71) = 5.14, *p* < .001, *CI* 95% [.13, .29]) but age was non-significant (*β* = .04, *t*(124.45) = .13, *p =* .90, *CI* 95% [-.55, .63]). These results suggest that, as age increases, depression symptoms also increase in both sexes, with the effect being more pronounced at older ages.

In a separate model, we tested the moderating effect of sex on the association between age and depressive symptoms. The interaction terms were not statistically significant in predicting depression symptoms (sex * linear age: *β* = -.32, *t*(112.88) = -1.59, *p* = .11, *CI* 95% [-.71, .08]; sex * quadratic age: *β* = -.04, *t*(101.19) = -.52, *p* = .60, *CI* 95% [-.20, .12]). This suggests that males and females show similar age-related patterns of increase in depression over time. See *Supplementary Figure 1* for a depiction of the associations between non-linear age and depression by sex.

*
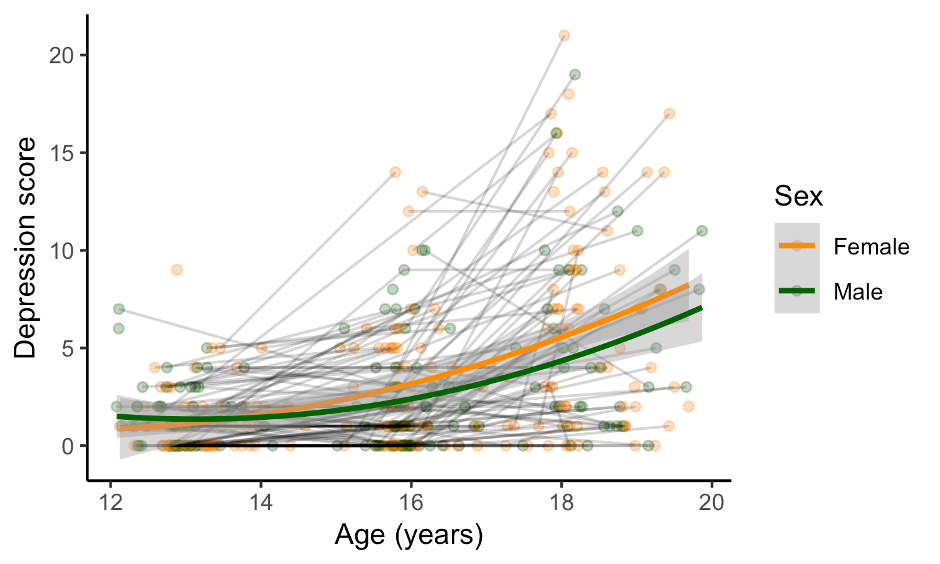
*

*Supplementary Figure 1.* Non-linear age and sex effects on depression scores.

### ***P3 amplitude on depression symptom severity and diagnosis***

### *1. P3 amplitude effects on continuous depression symptom:* Including the between- and within-person effects of P3 as random slopes did not improve model fit in congruent (fixed slopes: *AIC* = 2,029.94, *BIC* = 2,049.40, *LLRT* = -1,009.97; random slopes: *AIC* = 2,040.87, *BIC* = 2,079.79, *LLRT* = -1,010.43; model comparison: *χ2*(5) = 0, *p* = 1) and incongruent trials (​​fixed slopes: *AIC* = 2,025.42, *BIC* = 2,044.88, *LLRT*= -1,007.71; random slopes: *AIC* = 2,036.07, *BIC* = 2,074.99, *LLRT*= -1,008.04; model comparison: *χ2*(5) = 0, *p* = 1). Consequently, within- and between-person P3 amplitudes were modeled as fixed slopes in all models.

### The within- and between-person effects of P3 amplitude were not significantly associated with depression on congruent trials (within-person P3: *β* = -.20, *t*(238.22) = -1.66, *p* = .10, *CI* 95% [-.43, .04]; between-person P3: *β* = -.06, *t*(201.37) = -.86, *p* = .39, *CI* 95% [-.18, .07]) but the within person effect of P3 amplitude was significantly associated with depression on incongruent trials (within-person P3: *β* = -.29, *t*(243.06) = -2.70, *p* = .01, *CI* 95% [-.50, -.08]; between-person P3: *β* = -.03, *t*(200.97) = -.50, *p =* .62, *CI* 95% [-.16, .10]). These results suggest that, when age- and sex-related changes in depression are not accounted for, within-person increase in P3 amplitude predicts reduction in depressive symptoms on incongruent trials, whereas between-person differences in P3 amplitude do not predict depression.

### *2. P3 amplitude effects on depression diagnosis:* The within- and between-person effects of P3 amplitude were not significantly associated with depression on congruent trials (within-person P3: *β* = -.13, *z* = -1.15, *p* = .25, *CI* 95% [-.34, .09]; between-person P3: *β* = -.07, *z* = -1.10, *p* = .27, *CI* 95% [-.19, .05]) but the within person effect of P3 amplitude was significantly associated with depression on incongruent trials (within-person P3: *β* = -.23, *z* = -2.27, *p* = .02, *CI* 95% [-0.42, -.03]; between-person P3: *β* = -.06, *z* = -.94, *p* = .35, *CI* 95% [-.18, .06].). These results suggest that, when age- and sex-related changes in depression diagnosis are not controlled for, increases in P3 amplitude within individuals on incongruent trials are associated with a significant decrease in the odds of meeting diagnostic criteria for depression. In contrast, between-person differences in P3 amplitude were not related to depression diagnosis.

### *3. Age, sex, within- and between-person P3 amplitude effects on continuous depressive symptoms:* Between- and within-person P3 amplitude were modeled as fixed effects and age (linear and quadratic terms) as random slopes. On congruent trials, sex (*β* = .13, *t*(125.89) = .40, *p* = .69, *CI* 95% [-.50, .76]), within- (*β* = -.01, *t*(99.48) = -.14, *p* = .89, *CI* 95% [-.13, .11]) and between-person P3 amplitude (*β* = -.04, *t*(139.51) = -.89, *p* = .37, *CI* 95% [-.13, .05]) were not significant predictors of depression. Both linear and quadratic age terms were significant predictors of depressive symptoms (linear age: *β* = .89, *t*(125.89) = 8.95, *p* = < .001, *CI* 95% [.69, 1.09]; quadratic age: *β* = .21, *t*(125.89) = 5.09, *p* = < .001, *CI* 95% [.13, .29]). These findings suggest that depressive symptoms increases with age, with the rate of increase accelerating as age increases, and within- and between-person P3 amplitude is not predictive of the age-related change in depression.

### On incongruent trials, sex (*β* = .13, *t*(124.39) = .42, *p* = .68, *CI* 95% [-.49, .76]), between- (*β* = -0.04, *t*(140.15) = -0.88, *p* = 0.38, *CI* 95% [-.13, .05]) and within-person P3 amplitude (*β* = -.01, *t*(102.82) = -.19, *p* = .85, *CI* 95% [-.12, .10]) were not significant predictors of depressive symptoms. Both linear and quadratic age terms were significant predictors of depressive symptoms (linear: *β* = .89, *t*(124.39) = 8.89, *p* = < .001, *CI* 95% [.69, 1.09]); quadratic: *β* = .21, *t*(124.39) = 5.08, *p* = < .001, *CI* 95% [.12, .29]). These results suggest that when age was included in the model, the effect of within-person P3 amplitude on depression on incongruent trials became non-significant, likely due to the shared variance between age and within-person P3 amplitude in predicting depressive symptoms. Age continued to be the strongest predictor of change in depressive symptoms.

### *4. Age, sex, within- and between-person P3 amplitude effects on depression diagnosis:* The model fit indices indicated that including a quadratic age term as a covariate did not improve model fit (congruent - linear age only: *AIC* = 225.23, *BIC* = 247.56, *LLRT* = -106.62; linear and quadratic age: *AIC* = 225.65, *BIC* = 251.69, *LLRT* = -105.83; model comparison: *χ2*(1) = 1.58, *p* = .21; incongruent - linear age only: *AIC* = 225.02, *BIC* = 247.34, *LLRT* = -106.51; linear and quadratic age: *AIC* = 225.23, *BIC* = 251.27, *LLRT* = -105.61; model comparison: *χ2*(1) = 1.79, *p* = .18), therefore only the linear age term was modeled.

### On congruent trials, sex (*β* = -1.53, *z* = -1.92, *p* = .06, *CI* 95% [-3.08, 0.03]), within- (*β* = .04, *z* = .25, *p* = .80, *CI* 95% [-.29, .38]) and between-person P3 amplitude (*β* = .05, *z* = .49, *p* = .62, *CI* 95% [-0.15, 0.25]) were not significant predictors of depression diagnosis. Age, however, remained a statistically significant predictor of depression diagnosis (*β* = .87, *z* = 3.67, *p* = < .001, *CI* 95% [.41, 1.34]). On incongruent trials, sex (*β* = -1.51, *z* = -1.88, *p* = .06, *CI* 95% [-3.08, .06]), within- (*β* = -.08, *z* = -.52, *p* = .60, *CI* 95% [-.37, .21]) and between-person P3 amplitude (*β* = .04, *z* = .42, *p* = .67, *CI* 95% [-.16, .24]) were not statistically significant predictors of depression diagnosis, but age remained statistically significant (*β* = .85, *z* = 3.56, *p* < .001, *CI* 95% [.38, 1.32]).

### These results suggest that, regardless of trial type and after controlling for age- and sex-related changes in depression diagnosis, within- and between-person P3 amplitudes were significantly associated with the odds of meeting diagnostic criteria for depression. Age, however, remained a significant predictor of depression diagnosis on both congruent and incongruent trials.

**Section 3**

Supplementary Table 4

*Model comparison indices for Models 1-7.*

|  |  | **Congruent Trials** | | | **Incongruent Trials** | | |
| --- | --- | --- | --- | --- | --- | --- | --- |
| Model | Within-person variable | Random intercept only | Random intercept and slope | Model comparison | Random intercept only | Random intercept and slope | Model comparison |
| *Developmental Processes* | Age | AIC^7^ = 1936.28 | AIC = 1933.61 | *χ*2 = 6.67 | AIC = 1997.68 | AIC = 1998.16 | *χ*2 = 3.52 |
|  |  | BIC^8^ = 1955.96 | BIC = 1961.16 | *p* = .036 | BIC = 2017.35 | BIC = 2025.7 | *p* = .172 |
|  |  | LLRT^9^ = -963.14 | LLRT = -959.81 |  | LLRT = -993.84 | LLRT = -992.08 |  |
| *Depression 1* | Within-person depression, CBCL^1^/ASR^2^ | AIC = 1887.07 | AIC = 1890.29 | *χ*2 = .78 | AIC = 1958.71 | AIC = 1960.23 | *χ*2 = 2.48 |
|  |  | BIC = 1906.52 | BIC = 1917.53 | *p* = .678 | BIC = 1978.16 | BIC = 1987.47 | *p* = .29 |
|  |  | LLRT = -938.53 | LLRT = -938.14 |  | LLRT = -974.35 | LLRT = -973.11 |  |
| *Depression 2* | Within-person depression and age, CBCL/ASR | AIC = 1857.42 | AIC = 1860.34 | *χ*2 = 7.08 | AIC = 1918.2 | AIC = 1924.12 | *χ*2 = 4.08 |
|  |  | BIC = 1884.67 | BIC = 1907.04 | *p* = .214 | BIC = 1945.44 | BIC = 1970.82 | *p* = .538 |
|  |  | LLRT = -921.71 | LLRT = -918.17 |  | LLRT = -952.1 | LLRT = -950.06 |  |
| *Depression 3* | Depression diagnosis, KSADS^3^/SCID^4^ | AIC = 1596.59 | AIC = 1600.06 | *χ*2 = .52 | AIC = 1661.95 | AIC = 1661.59 | *χ*2 = 4.36 |
|  |  | BIC = 1611.47 | BIC = 1622.38 | *p* = .769 | BIC = 1676.83 | BIC = 1683.91 | *p* = .113 |
|  |  | LLRT = -794.29 | LLRT = -794.03 |  | LLRT = -826.98 | LLRT = -824.79 |  |
| *Depression 4* | Age and depression diagnosis, KSADS/ SCID | AIC = 1566.09 | AIC = 1568.86 | *χ*2 = 7.24 | AIC = 1623.77 | AIC = 1627.68 | *χ*2 = 6.09 |
|  |  | BIC = 1588.41 | BIC = 1609.78 | *p* = .204 | BIC = 1646.09 | BIC = 1668.6 | *p* = .298 |
|  |  | LLRT = -777.05 | LLRT = -773.43 |  | LLRT = -805.88 | LLRT = -802.84 |  |
| *Anxiety 1* | Within-person anxiety, SCA(A)RED^5/6^ | AIC = 1843.35 | AIC = 1843.87 | *χ*2 = 3.48 | AIC = 1914.32 | AIC = 1915.04 | *χ*2 = 3.28 |
|  |  | BIC = 1862.67 | BIC = 1870.91 | *p* = .175 | BIC = 1933.64 | BIC = 1942.08 | *p* = .194 |
|  |  | LLRT = -916.68 | LLRT = -914.93 |  | LLRT = -952.16 | LLRT = -950.52 |  |
| *Anxiety 2* | Age and within-person anxiety, SCA(A)RED | AIC = 1809.42 | AIC = 1809.09 | *χ*2 = 10.33 | AIC = 1868.18 | AIC = 1872.8 | *χ*2 = 5.38 |
|  |  | BIC = 1836.46 | BIC = 1855.45 | *p* = .066 | BIC = 1895.23 | BIC = 1919.17 | *p* = .372 |
|  |  | LLRT = -897.71 | LLRT = -892.54 |  | LLRT = -927.09 | LLRT = -924.4 |  |

***Note.*** ^1^CBCL - Child Behavior Checklist; ^2^ASR - Adult Self Report; ^3^KSADS - Kiddie Schedule for Affective Disorders and Schizophrenia; ^4^SCID - Structured Clinical Interview for DSM Disorders; ^5^SCARED - Screen for Child Anxiety Related Emotional Disorders; ^6^SCAARED - Screen for Adult Anxiety Related Emotional Disorders; ^7^AIC - Akaike Information Criterion; ^8^BIC - Bayesian Information Criterion; ^9^LLRT - Log-Likelihood Ratio Test.

We used the following criteria to evaluate the AIC, BIC, and Log-likelihood Ratio Test (LLRT) estimations: smaller AIC and BIC values indicate better model fit, with a difference of 2 or more considered the threshold for deeming one model superior to another. For the Log-likelihood Ratio Test, we compared the likelihoods of nested models, with the test statistic following a Chi-squared distribution. A significant p-value (<.05) from the Chi-squared test suggests that the more complex model provides a significantly better fit to the data than the simpler model.
